# Paediatric anaemia in rural Kenya and the role of travel time to emergency care services

**DOI:** 10.3389/fepid.2025.1578522

**Published:** 2025-05-15

**Authors:** Moses M. Musau, Cynthia Khazenzi, Samuel Akech, Evans Omondi, Emelda A. Okiro, Robert W. Snow, Peter M. Macharia, Alice Kamau

**Affiliations:** ^1^ Epidemiology and Demography Department, Kenya Medical Research Institute (KEMRI)—Wellcome Trust Research Programme, Nairobi, Kenya; ^2^ Institute of Mathematical Sciences, Strathmore University, Nairobi, Kenya; ^3^ African Population and Health Research Center, Nairobi, Kenya; ^4^ Centre for Tropical Medicine and Global Health, Nuffield Department of Clinical Medicine, University of Oxford, Oxford, United Kingdom; ^5^ Department of Public Health, Institute of Tropical Medicine Antwerp, Antwerp, Belgium; ^6^ Department of Clinical Sciences, Liverpool School of Tropical Medicine, Liverpool, United Kingdom

**Keywords:** anaemia, hospitalisation, access, travel time, model-based geostatistics

## Abstract

**Background:**

Access to emergency care (EC) services is crucial for severe anaemia outcome. Limited information exists on the association between travel times to EC services and the presentation and severity of anaemia upon hospital admission. Here, we investigate the association between travel time and presentation of severe anaemia (compared to mild/moderate anaemia) at admission in western Kenya.

**Methods:**

Data from January 2020 to July 2023 from Busia County Referral Hospital were assembled for paediatric admissions aged 1–59 months residing in Busia County. Travel time from a patient's village to the hospital was calculated using a least cost path algorithm. Anaemia severity was categorised as mild (Hb ≥ 7–<10 g dl^−1^), moderate (Hb ≥ 5–<7 g dl^−1^) and severe (Hb < 5 g dl^−1^). We fitted a geostatistical model accounting for covariates to estimate the association between travel times to EC services and severe anaemia presentation.

**Results:**

Severe anaemia admissions had the highest median travel time of 36 min (IQR: 25,54) (*p*-value: <0.001). Compared to children living within a 30 min travel time to the hospital, the adjusted odds ratio (AOR) of severe anaemia presentation relative to mild/moderate anaemia was 2.44 (95% CI: 1.63–3.55) for those residing within 30-59 min. For travel times of 60–89 min, the AOR was 3.55 (95% CI: 1.86–6.10) and for ≥90 min, the AOR was 3.41 (95% CI: 1.49–7.67).

**Conclusion:**

Travel time is significantly associated with the severity of paediatric anaemia presentations at hospitals. Addressing disparities in travel times such as strategic bolstering of lower-level facilities to offer EC services, is crucial for implementing new interventions and optimizing existing hospital-linked interventions to enhance healthcare delivery.

## 1 Introduction

Anaemia is a global public health problem (1, 2). In sub-Saharan Africa (SSA), it is estimated that over 43 million children under the age of five in the community suffer from a haemoglobin level (Hb) < 11 g dl^−1^ each year (3). The causes of anaemia in these children are manifold (4, 5), including nutritional deficiencies (6, 7), inherited haemoglobinopathies (8, 9) and the constant threats posed by bacteraemia (10) or parasitic infections, notably helminths (11) and malaria (12, 13).

Clinical severe anaemia, defined as Hb < 5 g dl^−1^, is a life-threatening event requiring emergency hospital care and blood transfusion (14, 15). Survival in hospital depends critically on the severity of the presenting illness, timeliness and reliability of blood transfusion, and quality of supportive care (16, 17). Policy recommendations emphasize the need to improve the quality of emergency care (EC) services (18), ensure the availability and safety of blood for transfusion (19–21), and optimise the management of children post-discharge to prevent re-admission and post-discharge mortality (22–24).

In Kenya, the prevalence of anaemia among children under the age of 5 years was estimated at 45.5% in 2020 (25). The national guidelines recommend blood transfusion for all severe anaemia patients irrespective of other clinical manifestations (26). However, the country not only falls short in the required blood supply (27), but blood transfusion EC services are also only available in higher-level hospitals, which are few and primarily located in urban areas (28, 29). This introduces accessibility inequities to this essential EC service, affecting the management of severe anaemia in rural communities that need prompt interventions and hospital-linked aftercare.

Understanding the increased risk of poor outcomes resulting from late presentation or prolonged distances/travel times to EC services is an essential component of hospital-based care policy and intervention (30). Studies have explored the relationship between distance/travel time and hospital outcomes in several African hospital settings, reporting an increased risk of adverse outcomes associated with prolonged distances/travel times. These include higher in-hospital paediatric mortality (31–34) and disease severity (35, 36). Consequently, various efforts have been undertaken to define travel time thresholds for different conditions, for example, in emergency obstetric care for maternal and neonatal health, the travel time threshold is often defined as within 2–3 h (37–39), while for emergency and trauma-related interventions, it is commonly referred to as the “golden hour” (40, 41). However, there is a paucity of information specifically related to anaemia and how travel time to EC services is associated with its presentation and severity at hospital.

Time to intervention administration is a key determinant of anaemia disease progression (16). Kiguli et al. (15) demonstrates that 90% of deaths due to severe anaemia occurred within 2.5 h of admission among admissions who do not receive blood transfusion. However, with EC services being limited to only a few high-level hospitals, severe anaemia patients might be forced to spend extended travel times to receive the recommended interventions. This phenomenon is known as distance decay in healthcare utilisation where a reduction in health service access is seen with an increase in distance or travel time (42, 43). Therefore, longer travel time has been associated with delayed care-seeking patterns (44, 45), which may result in patients presenting with more severe anaemia by the time they reach medical care.

In this study, we model travel time to hospital using a least cost path algorithm and explore its relationship with severe anaemia at admission, based on the hypothesis that anaemic patients who live in remote areas from the hospital are more likely to present with severe anaemia than those who live in nearby villages. Specifically, we utilise a Bayesian Model-Based geostatistical (MBG) framework accounting for covariates and residual spatial autocorrelation in anaemia burden to investigate the association between travel time and the likelihood of presenting with severe anaemia (over mild and moderate anaemia) at admission in a malaria endemic region in western Kenya. Our results show that scarcity of EC services for severe anaemia leads to (1) increased travel times among anaemia patients and (2) increased risk in the presentation of severe anaemia at admission. Lower-level facilities can be supported to provide EC services for severe anaemia patients to mediate the observed inequities in travel times to EC services.

## 2 Methods

### 2.1 Study area and context

**Figure 1:**
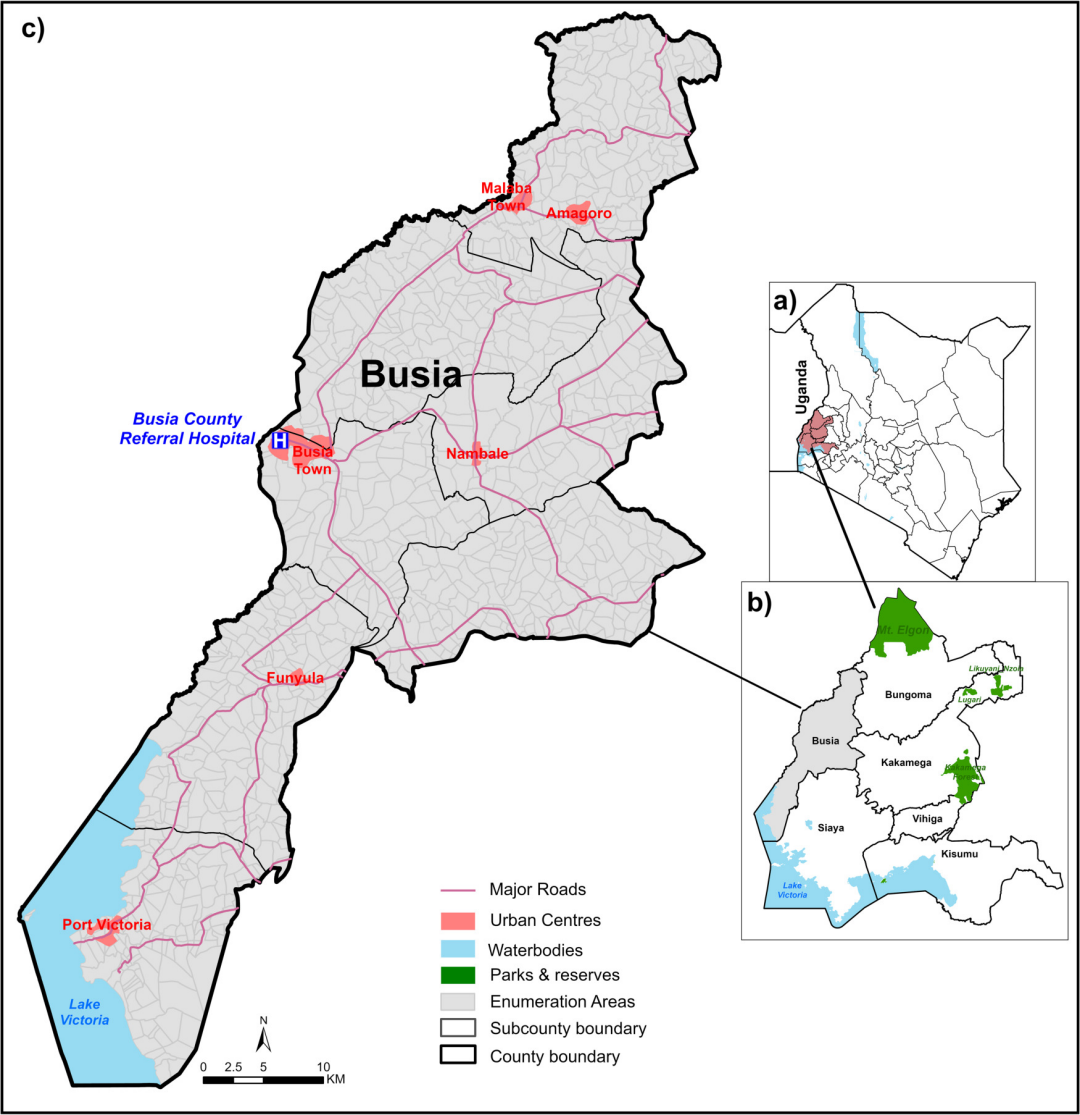
Study area: **panel (a):** Kenya counties highlighting western region. **Panel (b):** Busia County and neighbouring western Kenya counties. **Panel (c):** Enumeration areas of Busia County.

This study was a retrospective analysis of data collected at Busia County Referral Hospital (BCRH) in western Kenya (Figure 1). This hospital primarily serves residents of Busia County, approximately 893,681 residents (46), and is the referral point for lower-level facilities requiring specialised clinical care with the right personnel, treatment and/or equipment including blood transfusion services (28). Access to BCRH is possible via a 1,600 km road network comprising tarmac, gravel and earth surface roads (47) (Figure 1). Since 2013, BCRH has been part of a Clinical Information Network (CIN), which aims to improve the quality of inpatient paediatric care through systematic collection and use of clinical data (48–50). In 2019, surveillance efforts at BCRH were enhanced to include more routine malaria and haemoglobin testing, as part of the RTS,S malaria vaccine implementation study (51).

Busia County (a subnational unit of decision making) is characterised by sustained high intensity malaria transmission (52, 53) and a high burden of severe malaria anaemia presenting to the hospital (54, 55). Additionally, it is one of the areas in Kenya with the highest burden of soil-transmitted helminths and schistosomiasis among school-aged children (56). According to the 2022 national demographic and household survey, 15% of children aged under 5 years in Busia County were stunted and 6.3% were wasted (57). There are no empirical estimates of the gene frequency of haemoglobin S (Hb S) in Busia County; however, in the neighbouring counties (and Uganda), sickle cell trait (HbAS) was reported to be between 16%–19% (58–60). A recent malaria household survey in 2020 reported that the community anaemia prevalence among children aged 6–59 months in Busia County was 26.1% (Hb < 11 g dl^−1^), with moderate anaemia at 35.3% (Hb ≥ 7 to <11 g dl^−1^) and severe anaemia at 1.1% (Hb < 7 g dl^−1^) (25).

### 2.2 Data collection

The paediatric ward surveillance system at BCRH utilises a Paediatric Admission Record (PAR) form designed to standardise the documentation of routine clinical, laboratory and other investigations in the hospital. Nurses and clinicians on duty use the PAR to document patient details at admission, during hospitalisation and at discharge (or death). A trained data clerk then abstracts all data from the patient file in REDCap (49). The abstracted data includes patients' demographics, residence details, anthropometric measurements, medical history, clinical examinations, laboratory tests ordered and their results, prescribed treatments and the final discharge diagnosis. Haemoglobin (Hb) concentrations were measured from capillary bloods at admission using a Hematology Analyzer (Coulter Counter), with results recorded in g dl^−1^. Final diagnoses were reviewed to identify children with underlying conditions including genetic and congenital abnormalities, HIV, tuberculosis, trauma, burns, accidental poisoning, animal/snake/insect bites, epilepsy and carcinomas.

Residence details obtained at admission included various administrative sub-divisions including sub-county, location, sub-location, village name, nearest health facility, nearest markets and nearest school (nearness was as perceived by the respondent). This information was used to locate each child's residence using mapped national census enumeration areas (EA), which are equivalent to small areas representing “villages” of approximately 100 households (46).

#### 2.2.1 Anaemia definition

The Hb level was adjusted for altitude as recommended by WHO (61, 62). The adjusted Hb concentration was used to define three levels of clinically important hospitalised anaemia: mild (Hb ≥ 7 to <10 g dl^−1^), moderate (Hb ≥ 5 to <7 g dl^−1^) and severe (Hb < 5 g dl^−1^) (15).

#### 2.2.2 Data inclusion and exclusions

For this study, data covering January 2020 to July 2023 (42 surveillance months) for all children aged 1–59 months who were residents of Busia County were extracted (*n* = 4,361) (Supplementary Figure S1). This period represents a time when more intensive haemoglobin surveillance and detailed patient residence details were introduced (51). The admission cases included in this study were selected to provide a focused understanding of the relationship between travel time to hospital and the severity of anaemia in children. Specifically, cases related to animal/snake/insect bites, burns, malignancy/congenital abnormalities, poisoning, surgery, and trauma/accidents were excluded from the analysis (*n* = 665) as these conditions may not be directly linked to the place of residence, as they are likely to have occurred elsewhere (Supplementary Figure S1). Additionally, 264 admissions were excluded because Hb results were not available, including 27 deaths which included 19 deaths on arrival. There were no differences in any characteristics between the children included in this analysis and those excluded (Supplementary Table S1). Furthermore, children who were not anaemic (Hb ≥ 10 g dl^−1^) were also excluded from the analysis (*n* = 1,245).

#### 2.2.3 Travel time to hospital

The time taken to travel from the patient's village (EA) of residence was calculated using a least cost path modelling approach in AccessMod software (alpha version 5.8.0) (63). Briefly, road network, land use/cover, water bodies and protected areas (Supplementary Notes 2) were merged to create a friction surface (Supplementary Figure S2). Different speeds and modes of travel (walking, motorcycle and vehicle transport) were assigned to the friction surface based on previous literature (64–66) (see Supplementary Table S2). The friction raster surface was then combined with the least cost path algorithm (67) and the location of BCRH to obtain a travel time raster indicating for each pixel (grids of 12.5 m by 12.5 m) the lowest travel to time to BCRH. Average travel times for each EA were extracted from the travel time raster surface and assigned to all paediatric admissions originating from the respective EAs (Supplementary Figure S3).

### 2.3 Anaemia admissions spatial patterns

To visualise how travel time to EC services varies with anaemia admissions, anaemia admissions rates per 1,000 children were calculated for each EA over the 42-month period using population estimates for children under five years old. The probability of an EA having an anaemia admission is influenced by the at-risk population density, assuming homogeneous risk. Annual population estimates (unconstrained UN-adjusted) during the surveillance period were derived from WorldPop (https://www.worldpop.org/), which provides a 100 × 100 m gridded population density (68) and used to obtain the total under 5 population at risk denominator. Travel time was divided into 15-minute bands, and the admissions rates per 1,000 children over the 42-month period categorised by anaemia severity (mild, moderate, severe) within each band were calculated. Our estimated admission rates, however, represent a minimum measure of the true community burden of anaemia, as anaemia events may have occurred outside BCRH, with the child either recovering or dying at home. The 15 min travel time cut-offs were specifically chosen to visualise distance decay patterns in admission rates across the anaemia categories at a more granular level.

### 2.4 Statistical analysis

#### 2.4.1 Descriptive statistics

We compared patient and community-level factors across the three anaemia classes (mild, moderate and severe) using Fisher's exact test for categorical variables and Kruskal–Wallis rank sum test for continuous variables. Additionally, a 95% Confidence Interval (CI) for proportions was estimated using Wilson test for categorical variables. The median and interquartile range (IQR) were calculated for continuous variables.

Patient-level factors included age, categorised into yearly age groups, and gender. Mid-upper arm circumference (MUAC) was recorded, and nutritional status was defined as well nourished (Z-score > −1), mildly malnourished (−2 <Z-score ≤ −1), moderately malnourished (−3 < Z-score ≤ −2) and severely malnourished (Z-score ≤ −3) (69). Children who received BCG, Penta 1 and Penta 3 vaccines were defined as having a good vaccination history. We also included whether malaria was diagnosed at discharge, history and/or diagnosis of Sickle Cell Disease (SCD), admission day (weekday/weekend) and season (wet/dry); season was associated with rainfall data with wet season typically occurring from April to June and from October to December. These patient-level factors were selected because they have been identified as potential confounders in the literature (7, 13, 33, 55, 70, 71).

Given the significance of malaria in hospitalised anaemia cases in Busia County (54, 55), we adjusted for community-level variations in malaria infection risk using an EA-specific prediction of *Plasmodium falciparum* prevalence in children aged 2–10 years (*Pf*PR_2–10_). These predictions were derived from a temporal-spatial model based on malaria prevalence estimates from surveys [details provided in Alegana et al. (53)]. Admissions were assigned the average *Pf*PR_2–10_ for their respective EA over the four years preceding the surveillance period (Supplementary Notes 3 and Supplementary Figure S4).

Residence type (urban or rural) was also included as these areas differ in terms of ease of access to EC services, including available transportation options. Each EA was classified as either urban or rural based on the average nighttime light (NTL) values from the 2019 satellite image by the Defense Meteorological Satellite Program (DMSP) Operational Linescan System (OLS) (https://eogdata.mines.edu/products/dmsp/). NTL was used as a proxy for both urbanicity and economic status (72, 73). EAs with NTL > 0 were classified as urban, while those with NTL = 0 were deemed rural.

#### 2.4.2 Geostatistical modelling

The analysis focused on assessing the association of travel time to EC services on the presentation of severe anaemia compared to both mild and moderate anaemia. The outcome variable was categorised into two groups: severe anaemia and other (a combination of mild and moderate anaemia admissions). Travel time was categorized into 30 min intervals, considering the current 1–2 h threshold for emergency services and the high mortality rate within 2.5 h of admission for severe anaemia. Currently, there are no clear travel time thresholds specific to anaemia, except time-to-blood transfusion after admission, for example, Thomas et al. (74) and Maitland et al. (16) investigate differences in the risk of mortality in the case of immediate blood transfusion or delayed transfusion. Therefore, the 30 min interval [routinely used in general emergency care thresholds (75–77)] used here reflects the acute nature of severe anaemia before hospitalisation. Our aim was to test the robustness of existing policy thresholds and provide a conservative travel time estimate for acute severe anaemia. All covariates were modelled as categorical variables; *Pf*PR_2–10_ was modelled as a continuous variable. A univariate frequentist logistic regression model was used to select the covariates to be included in the final model. The selection was based on *p*-value threshold of *P* < 0.2 (Supplementary Notes 4 and Supplementary Table S3).

We assessed the presence of spatial autocorrelation while accounting for potential confounders using a variogram (Supplementary Notes 5 and Supplementary Figure S5). Given spatial autocorrelation, we employed a Bayesian Model-based geostatistics (MBG) framework (78) with a focus on explanatory modelling and not predictive modelling (79, 80). This approach allowed us to account for potential confounders and residual spatial autocorrelation when assessing the role of travel time to EC services on the presentation of severe anaemia.

All covariates were defined as fixed effects denoted by $\beta_{j}x_{j}$, where $x_{j}$ is the set of covariates (including travel time) and $\beta_{j}$ the set of corresponding coefficients. The random effect was defined as $\omega (s_{k})$ and $\epsilon_{i}$ representing the residual spatial variation with respect to EA $s_{k}$ and margin of error, respectively. Let $Y_{ik}$ be an admission *i* from village $s_{k}$ such that $Y_{ik}=1$ if the admission had severe anaemia and $Y_{ik}=0$ if the admission had mild/moderate anaemia and let $\pi_{ik}$ be the probability of an admission having severe anaemia. Then, $Y_{ik}∼\;Bernoulli\;(\pi_{ik})$ and is computed as

$$logit\;(\pi_{ik})=\beta_{0}+\overset{p}{\underset{\;j=1}{\sum }}\beta_{j}x_{j}+\omega (s_{k})+\epsilon_{i}\;$$

where $\omega (s_{k})∼\;N(0,\sigma_{\omega }^{2})$ and $\sigma_{\omega }^{2}$ was assumed to follow the Matèrn covariance structure such that for two EAs $s_{i}$ and $s_{j}$, $\sigma_{\omega }^{2}(s_{i},s_{j})=\frac{\sigma^{2}}{2^{\nu -1}\Gamma (\nu )}(\kappa h{)}^{\nu }K_{\nu }(\kappa h)$ where *h* is the Euclidean distance between centroids for EAs $s_{i}$ and $s_{j}$, *v* s the smoothness parameter, κ is the range parameter, $\sigma^{2}$ is the marginal variance, $K_{v}$ is the modified Bessel function of the second kind, and Γ(⋅) denotes the gamma function. The model was fitted using the Integrated Nested Laplace Approximation and Stochastic Partial Differential Equation (INLA-SPDE) framework with non-informative priors (81–84). Given the continuous nature of the surveillance during the study period, it is possible that some children were readmitted at different time points. However, we could not differentiate between initial admissions and readmissions due to lack of a unique admission identifier in the data, thus, each admission was treated as an independent observation in all the analysis.

All analyses were conducted in R software version 4.4.0 (2024-04-24 ucrt) using “gtsummary” and “INLA” packages for descriptive and goestatistical modelling, respectively. Map visualisations were done on ArcMap 10.8.2 (ESRI Inc., Redlands, CA, USA).

## 3 Results

### 3.1 Description of patient and community-level characteristics

The analysis included 2,187 admissions with Hb less than 10 g dl^−1^ aged 1–59 months residing in Busia County for the period January 2020 – July 2023: 1,151 (52.6%) were classified as mild anaemia, 402 (18.4%) as moderate anaemia and 634 (29.0%) as severe anaemia. Severe anaemia admissions had the highest median travel time of 36 min (IQR: 25,54), followed by moderate anaemia admissions with a median travel time of 26 min (IQR: 9,43) and mild anaemia admissions had the shortest median travel time of 17 min (IQR: 6,33) (*p*-value: < 0.001) (Table 1). Only 34.9% of severe anaemia admissions were within 30 min of travel time to BCRH compared to 55.7% and 71.8% of moderate and mild anaemia admissions, respectively (*p*-value: < 0.001) (Table 1). The proportion of admissions within 30–59 min of travel time was 23.0% for severe anaemia, 34.3% for moderate anaemia, and 47.6% for mild anaemia. Cumulatively, within the 1-hour travel time to BCRH (including both within 30 min and 30–59 min), 82.5% of severe anaemia admissions occurred, compared to 90.0% for moderate anaemia and 94.8% for mild anaemia admissions (*p*: <0.001) (Table 1).

**Table 1:** Characteristics of paediatric anaemia admissions to busia county referral hospital January 2020-July 2023.

Characteristic	Overall	Mild (Hb ≥ 7 to < 10 g dl^−1^)	Moderate (Hb ≥ 5 to < 7 g dl^−1^)	Severe (Hb < 5 g dl^−1^)	*p*-value
	N (%, 95% CI)	N (%, 95% CI)	N (%, 95% CI)	N (%, 95% CI)	
Total: N (%)	2,187 (100%)	1,151 (52.6%)	402 (18.4%)	634 (29.0%)	
Travel Time (mins): Median (IQR)	25 (9, 41)	17 (6, 33)	26 (9, 43)	36 (25, 54)	<0.001
Travel Time Classes (mins)					
<30	1,271 (58.1%; 56–60%)	826 (71.8%; 69–74%)	224 (55.7%; 51–61%)	221 (34.9%; 31–39%)	<0.001
30–59	705 (32.2%; 30–34%)	265 (23.0%; 21–26%)	138 (34.3%; 30–39%)	302 (47.6%; 44–52%)	
60–89	160 (7.3%; 6.3–8.5%)	37 (3.2%; 2.3–4.4%)	36 (9.0%; 6.4–12%)	87 (13.7%; 11–17%)	
>= 90	51 (2.3%; 1.8–3.1%)	23 (2.0%; 1.3–3.0%)	4 (1.0%; 0.32–2.7%)	24 (3.8%; 2.5–5.7%)	
Age Categories (yrs)					
<1	624 (28.5%; 27–30%)	388 (33.7%; 31–37%)	100 (24.9%; 21–29%)	136 (21.5%; 18–25%)	<0.001
1	653 (29.9%; 28–32%)	401 (34.8%; 32–38%)	116 (28.9%; 25–34%)	136 (21.5%; 18–25%)	
2	362 (16.6%; 15–18%)	164 (14.2%; 12–16%)	69 (17.2%; 14–21%)	129 (20.3%; 17–24%)	
3	279 (12.8%; 11–14%)	112 (9.7%; 8.1–12%)	60 (14.9%; 12–19%)	107 (16.9%; 14–20%)	
4	269 (12.3%; 11–14%)	86 (7.5%; 6.1–9.2%)	57 (14.2%; 11–18%)	126 (19.9%; 17–23%)	
Gender: Female	902 (41.2%; 39–43%)	467 (40.6%; 38–43%)	165 (41.0%; 36–46%)	270 (42.6%; 39–47%)	0.7
Nutrition Status					
Well nourished	833 (41.6%; 39–44%)	446 (43.7%; 41–47%)	159 (41.8%; 37–47%)	228 (37.8%; 34–42%)	0.06
Mildly Malnourished	703 (35.1%; 33–37%)	344 (33.7%; 31–37%)	126 (33.2%; 28–38%)	233 (38.6%; 35–43%)	
Moderately Malnourished	287 (14.3%; 13–16%)	130 (12.7%; 11–15%)	65 (17.1%; 14–21%)	92 (15.3%; 13–18%)	
Severely Malnourished	180 (9.0%; 7.8–10%)	100 (9.8%; 8.1–12%)	30 (7.9%; 5.5–11%)	50 (8.3%; 6.3–11%)	
Vaccination history	2,055 (94.0%; 93–95%)	1,063 (92.4%; 91–94%)	388 (96.5%; 94–98%)	604 (95.3%; 93–97%)	0.003
(Received BCG, Penta 1 & Penta 3)					
Malaria Diagnosis	874 (40.0%; 38–42%)	386 (33.5%; 31–36%)	147 (36.6%; 32–42%)	341 (53.8%; 50–58%)	<0.001
Sickle Cell Disease (SCD)	351 (16.0%; 15–18%)	93 (8.1%; 6.6–9.8%)	107 (26.6%; 22–31%)	151 (23.8%; 21–27%)	<0.001
Admission Day: Weekday	1,716 (78.5%; 77–80%)	877 (76.2%; 74–79%)	319 (79.4%; 75–83%)	520 (82.0%; 79–85%)	0.015
Season: Wet	1,129 (51.6%; 50–54%)	589 (51.2%; 48–54%)	225 (56.0%; 51–61%)	315 (49.7%; 46–54%)	0.13
*Pf*PR_2−10_% Median (IQR)	33 (29, 46)	31 (28, 42)	33 (29, 47)	38 (30, 48)	<0.001
Residence type: Rural	1,548 (70.8%; 69–73%)	700 (60.8%; 58–64%)	295 (73.4%; 69–78%)	553 (87.2%; 84–90%)	<0.001

**Nutrition status**: *n* = 184 admissions were missing MUAC (131 in mild, 22 in moderate and 31 in severe anaemia classes).

For individual-level characteristics, severe anaemia admissions aged ≤1 year was significantly lower (43.0%) compared to mild (68.5%) and moderate (53.8%) anaemia admissions (Table 1). Good vaccination history was >90% across the three anaemia classes, with moderate anaemia having the highest proportion, 96.5%, followed by severe anaemia at 95.3% and mild anaemia at 92.4% (*p*-value: 0.003). The proportion of admissions with a malaria diagnosis was highest among severe anaemia admissions at 53.8% compared to 36.6% and 33.5% among moderate and mild anaemia classes, respectively (*p*-value: <0.001). Similarly, SCD was more prominent among severe and moderate anaemia admissions, 23.8% and 26.6% respectively, compared to only 8.1% among mild anaemia admissions (*p*-value: <0.001). Finally, a significantly higher proportion of severe anaemia admissions occurred on weekdays (82.0%) compared to mild and moderate anaemia classes (Table 1).

For community-level characteristics, severe anaemia admissions resided in areas with higher malaria transmission, with a median *Pf*PR_2–10_ of 38% (IQR: 30,48) compared to moderate and mild anaemia admissions with a median *Pf*PR_2–10_ of 33% (IQR: 29,47) and 31% (IQR: 28,42) respectively (*p*-value: <0.001) (Table 1). A significantly higher proportion of severe anaemia admissions resided in rural areas, 87.2% (*p*-value: <0.001) compared to the other anaemia classes (Table 1).

### 3.2 Anaemia admission spatial patterns by severity

**Figure 2:**
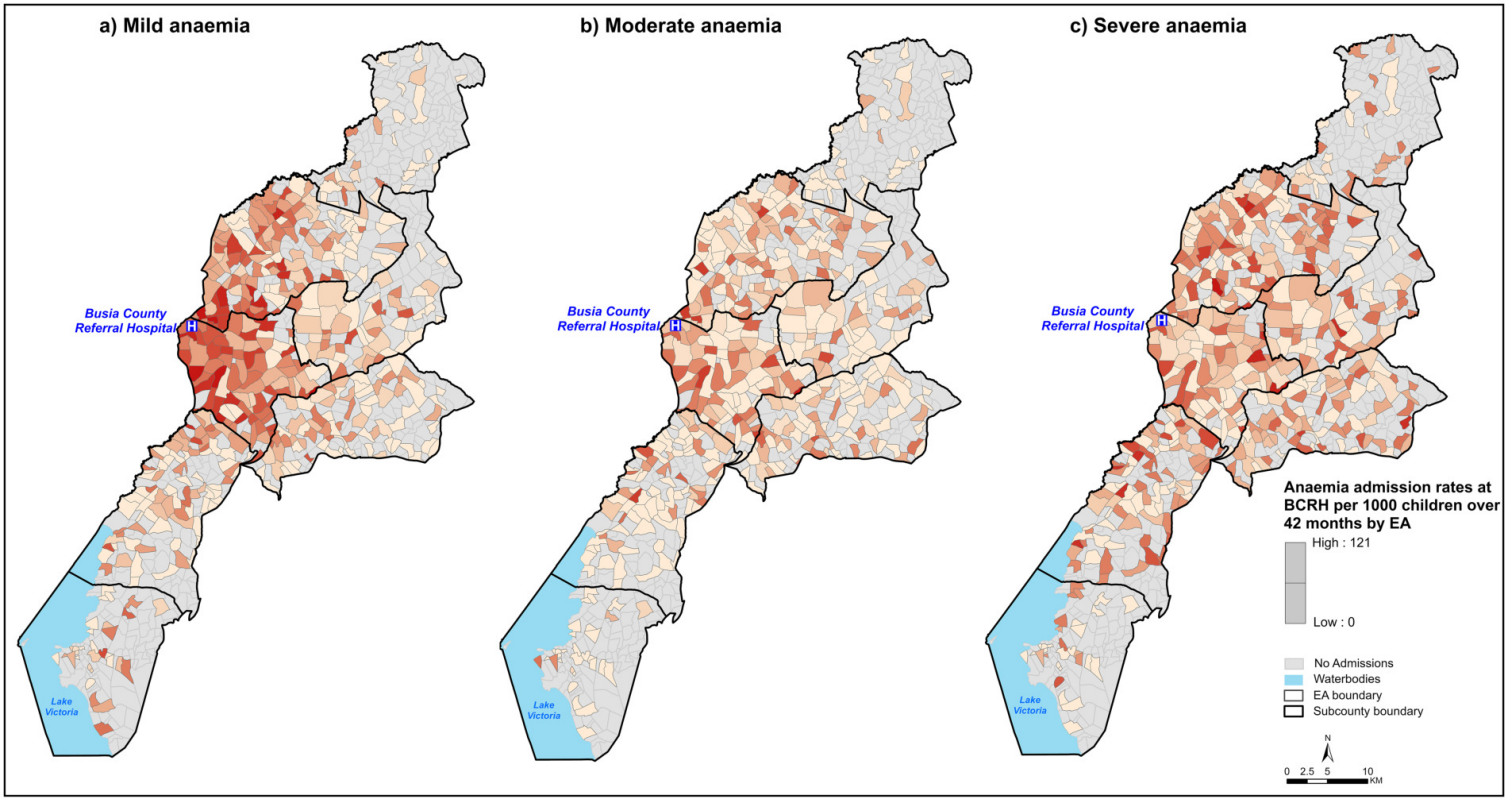
Spatial distribution of anaemia admission rates per 1,000 children aged under five years over 42 months of surveillance at BCRH by EA. EA shades represent anaemia admission rates where the darker the shade, the higher the admission rates. **Panel (a):** Mild anaemia, **Panel (b):** Moderate anaemia, **Panel (c):** Severe anaemia.

The median EA admissions rates per 1,000 persons over 42 surveillance months was 3.7 (IQR: 0.2, 38.6), 2.8 (IQR: 0.4, 49.7) and 3.7 (IQR: 0.3, 121) for severe, moderate and mild anaemia admissions, respectively, with markedly varying spatial patterns (Figure 2). Among mild anaemia admissions, EAs proximal to BCRH had high rates (darker shades) and a decreasing trend in rates is observed in EAs distal to BCRH (Figure 2, Panel a). A similar trend is observed among moderate anaemia admissions (Figure 2, Panel b). In contrast, there is no decreasing trend in admission rates among severe anaemia admissions, which were less concentrated around BCRH (Figure 2, Panel c).

**Figure 3:**
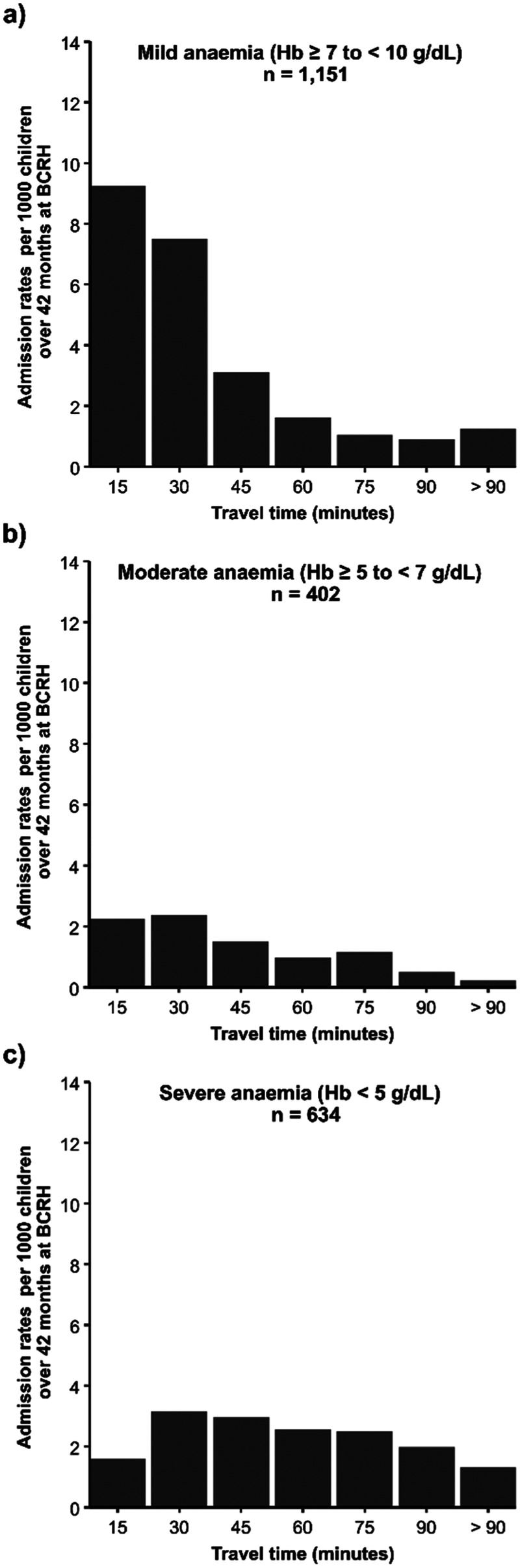
Anaemia admission rates per 1,000 children aged under five years over 42 surveillance months at BCRH by travel time bands in minutes. **Panel (a):** Mild anaemia, **Panel (b):** Moderate anaemia **Panel (c):** Severe anaemia.

Differences were observed in admission rates per 1,000 children over 42 surveillance months at BCRH by travel-time bands across the anaemia severity classes (Figure 3). Specifically, admission rates of mild anaemia admissions drop from ≈9 to ≈1.6 admissions per 1,000 persons/42 surveillance months for admissions within 15 min and those within 60 min of travel time to BCRH (Figure 3, Panel A). Rates of moderate anaemia admissions follow a similar pattern to mild anaemia, declining from ≈2.4 to ≈0.9 within 30 and 60 min of travel time to BCRH (Figure 3, Panel b). In contrast, for severe anaemia admissions, rate per 1,000 persons over 42 surveillance months increases from ≈1.5 to ≈3.2 for admissions within 15 min and those within 30 min and no marked decrease is observed at longer travel times (Figure 3, Panel c).

### 3.3 Travel time and severe anaemia presentation

In the univariate analysis, eight covariates were found to be statistically significant (*P* < 0.2): age, nutritional status, vaccination history, malaria diagnosis, SCD, admission day (weekday/weekend), community predicted *Pf*PR_2–10_ and residence type (Supplementary Notes 4 and Supplementary Table S3). Presence of residual spatial autocorrelation was evidenced by a variogram (Supplementary Notes 5 and Supplementary Figure S5).

After adjusting for age, nutritional status, vaccination history, malaria diagnosis, SCD, admission day (weekday/weekend), community predicted *Pf*PR_2–10_, residence type and residual spatial autocorrelation, travel time was significantly associated with increased odds of severe anaemia presentation over other anaemia classes (Table 2). The likelihood of severe anaemia relative to mild/moderate anaemia increased with longer travel times. Specifically, for children residing within 30–59 min to BCRH, the adjusted odds ratio (AOR) was 2.44 (95% CI: 1.63–3.55), 60–89 min the AOR was 3.55 (95% CI: 1.86–6.10) and ≥90 min the AOR was 3.41 (95% CI: 1.49–7.67) compared to children living within 30 min of travel time (see Supplementary Notes 6 and Supplementary Table S4 for AOR of covariates).

**Table 2:** Association between travel time to EC among children with severe anaemia compared to other (mild/moderate) anaemia.

Travel Time (mins)	N (%)	Crude OR (95% CI)	AOR (95% CI)
*<*30	1,271 (58.1%)	Ref	Ref
30–59	705 (32.2%)	3.56 (2.89–4.39)	2.44 (1.63–3.55)
60–89	160 (7.3%)	5.66 (4.02–8.00)	3.55 (1.86–6.10)
≥90	51 (2.3%)	4.22 (2.38–7.46)	3.41 (1.49–7.67)

## 4 Discussion

Travel time to hospital services is significantly associated with severity of anaemia presentation at admission (Table 1; Figure 3). Population-adjusted rates of admissions with severe anaemia indicate that these admissions are more distal to emergency care services than those with mild or moderate presenting anaemia (Figure 2). Importantly, adjusting for factors which may confound this relationship using a Bayesian MBG model confirmed that travel times greater than 30 min are associated with a 2-fold increase in the likelihood of severe anaemia compared to mild-moderate anaemia and a greater than 3-fold increased likelihood if greater than 60 min (Table 2).

The classic distance decay curve was observed in attendance (for moderate and mild anaemia) with increasing travel time (85). Treatment-seeking decays more rapidly after travel times of approximately 45 min (Figure 2), which emphasises the relevance of travel time in care-seekers' decision making process on where and if to seek care. More specifically, the observed decreasing trend in the spatial patterns for mild-moderate anaemia admission rates among distal EAs (Figure 2) can be attributed to the high density of lower-level health facilities (86) in the longer travel time locations that provide alternative treatment options as mild/moderate anaemia cases do not require blood transfusions. For mild-moderate anaemia admissions with longer travel times, this can also be interpreted as individual perception on quality of care at BCRH compared to existing alternatives. However, without additional data, we are unable to definitively conclude that this is the only explanation for the observed trend.

In contrast, the lack of a decreasing trend in admission rates among severe anaemia patients can be associated with a lack of treatment alternatives, as the high-level interventions required (blood transfusion) are only available at higher level facilities (28). As such, severe anaemia patients visit BCRH to compete for the existing EC blood transfusion services. In addition, the lower admission rates observed among EAs proximal to BCRH for severe anaemia patients may be indicative of prompt care-seeking behaviour, which prevents the progression of anaemia to a severe form.

Disease events that involve rapid haemolysis are critically dependent on the time taken to reach emergency care services, including blood transfusion. Prolonged travel times have been described as a deterrent to seeking care as distal populations from EC services tend to seek care only when the disease has progressed to a severe form (45, 87). Consequently, admissions with longer travel times present later at BCRH compared to those from more proximal areas. Furthermore, pre-referral care, notably for severe malaria, remains inadequate in Kenya (88), ambulance services in remote, rural areas are non-existent (89) thus, more communities in these areas may take longer to secure financial resources to pay for public transport. Our findings are consistent with other studies which have interrogated the role of travel time to hospital care and in-patient severe disease and mortality outcomes (31–34, 36), in summary, individuals who live further away from emergency care services have a poorer presentation and prognosis. We acknowledge that access to EC services is a more complex phenomenon and travel time alone is not the only determinant for improved health outcomes, for instance, improving road quality may have an impact on reduced travel times and consequently improved health outcomes. However, it is also essential to increase the availability of emergency care services in facilities closer to at-risk populations (Supplementary Notes 7 and Supplementary Figure S6) as evidenced in the implementation of community-based EC interventions for other illnesses (90, 91). Most of the overall anaemic admission population were children aged less than 3 years. However, 25% of children were aged 3 years or older, and highest among those with severe anaemia (36.8%) (Table 1). Furthermore, malaria was a final diagnosis in 40.0% of all anaemic admissions but significantly higher in those with severe anaemia (53.8%) (Table 1). As previously reported for Western Kenya, severe anaemia is a dominant disease phenotype for life-threatening malaria (54, 55). This is further supported by our observation that predicted malaria prevalence in the origin communities of those who presented with severe anaemia experienced higher levels of transmission (Table 1). Aside from malaria, it is notable that 16% of all anaemic admissions had a reported underlying diagnosis of Sickle Cell Disease, representing an important cause of anaemia requiring hospitalisation. In this area of Kenya, reported SCD was lowest in the mild anaemia class (8.1%) compared to both the moderate (26.6%) and severe (23.8%) anaemia classes. Both malaria and SCD are significant causes of moderate and severe hospital presentations of anaemia in this area and not restricted to the very young.

Identifying children with severe anaemia in hospital serves as the entry point for recently promoted post-discharge malaria chemoprevention strategies where children are provided monthly presumptive anti-malarial drugs (22, 24). An important consideration is how the intervention is provided (92). Most patients in our study would be more distal to centralised health services and thus more marginalised from facility-based chemoprevention and would depend on an effective, linked community-based aftercare service. However, more general post-discharge clinical review and investigation would continue to be compromised by distance. Similar constraints will apply to SCD patients related to access to hospital services for diagnosis, long-term care options and antimalarial and antimicrobial prophylaxis (93).

Busia County represents a rural setting in a low-and-middle income country with challenges in both the availability of EC services and limited access to the existing services. Consequently, the results of this work are generalisable to similar settings in sub-Saharan Africa that face similar EC challenges (90, 94). Furthermore, the implications can be generalised not only to anaemia specific interventions but also to other interventions such as post-discharge malaria chemoprevention programs where travel times/distance to care has been described as a barrier (24).

### 4.1 Strengths and limitations

Previous studies on the association between travel times to EC services and hospital health outcomes have not always adjusted for underlying drivers of the health outcomes (33, 34, 36) and/or residual spatial autocorrelation in the outcome at fine spatial resolution (31, 32). We have used a geostatistical model to overcome these inherent problems in explanatory modelling when assessing the role of travel time. We have achieved this by adjusting for the community-level factors such as malaria endemicity (*Pf*PR_2–10_) and residual spatial autocorrelation likely to characterise the association between travel time to EC and the severity of anaemia. This model allowed for a more robust definition of the increased risk of severe anaemia presentation at hospital with increasing travel time, independent of the contextual risk factors. Further, we used a robust and well-established approach to estimate travel time that accounted for a hybrid mode of transport adjusting for topography, travel barriers and travel speeds relative to the use of simplistic Euclidean distance or provider-to-population ratio (95).

While we were able to adjust the model for malaria and urbanisation at the level of the child's residence, we were not able to adjust for equivalent, high-spatial resolution community-level factors, such as socio-economic status (SES) and helminth prevalence. Socio-economic factors such as household income, guardian's education level and healthcare knowledge/practices have been shown to influence both healthcare seeking behaviours and disease severity at admission (32, 45, 96, 97). Not accounting for such factors may have confounded the observed association of travel time and disease severity as patients from lower income households may face additional barriers to timely healthcare access, independent of travel time. Furthermore, this may have led to bias where population from lower-income households consistently locate in an area with longer travel time and are characterized with distinct health seeking behaviours, preferences and knowledge compared to households located close to the main hospital. In addition, although we adjust for community-level malaria prevalence, this may introduce the risk of ecological fallacy, where the association observed at the community level does not necessarily apply to individuals within that community. We could not also define the aetiologies of the child's anaemia on admission without more comprehensive haematological profiling. Further, data on anaemic admissions from other competing facilities in the area was unavailable for analysis. As such, the study could not assess hospital competition to EC services which is an important factor to consider in assessing the significance of travel time to EC services (98). Consequently, the study represents a minimum measure of the true community burden of anaemia, as anaemia events may have occurred outside BCRH. Lastly, travel time was not based on observational data and did not account for seasonality or traffic delays, which, if present, affect travel speeds (99). However, as this is a predominately rural area, the effect of traffic is negligible and thus our estimates are plausible and representative of dry weather season (100).

## 5 Conclusion

Travel time to EC services is significantly associated with the degree of severity of paediatric anaemia at presentation in hospital. This study highlights how the scarcity of emergency care services for severe anaemia leads to increased travel times among severe anaemia patients. The findings from this study can be used to inform strategic bolstering of lower-level facilities to offer severe anaemia interventions to improve healthcare delivery in resource-limited settings.

## Data Availability

The datasets presented in this article are not readily available because Data for this report are under the primary jurisdiction of the Ministry of Health in Kenya. Enquiries about using the data can be made to the KEMRI-Wellcome Trust Research Programme Data Governance Committee. Requests to access the datasets should be directed to dgc@kemri-wellcome.org.
