# Supplementary material for: Paediatric anaemia in rural Kenya and the role of travel time to emergency care services
Source: Front Epidemiol. 2025 May 15;5:1578522. doi: 10.3389/fepid.2025.1578522 (PMC12119680; doi:10.3389/fepid.2025.1578522)
Supplement: Supplementary file 1 [file Datasheet1.pdf]

Supplementary Material

SUPPLEMENTARY NOTES 1

Characteristics of excluded admissions

Table S1: Characteristics of excluded children with underlying conditions/missing compared to those of the included children

**Characteristic**	**Underlying conditions (excluded) N (%, 95% CI)**	**Missing Hb (excluded) N (%, 95% CI)**	**Analysed dataset N (%, 95% CI)**
**Total: N(%)**	655 (100%)	264 (100%)	2,187 (100%)
**Travel Time (mins):** Median (IQR)	28 (13, 44)	17 (6, 36)	25 (9, 41)
**Travel Time Classes (mins)**<br><30<br>30-59<br>60-89<br>≥ 90	345 (51.9%; 48-56%)<br>243 (36.5%; 33-40%)<br>48 (7.2%; 5.4-9.5%)<br>29 (4.4%; 2.9-6.2%)	174 (65.9%; 60-72%)<br>61 (23.1%; 18-29%)<br>21 (8.0%; 5.0-12%)<br>8 (3.0%; 1.3-5.9%)	1,271 (58.1%; 56-60%)<br>705 (32.2%; 30-34%)<br>160 (7.3%; 6.3-8.5%)<br>51 (2.3%; 1.8-3.1%)
**Age Categories (yrs)**<br><1<br>1<br>2<br>3<br>4	131 (19.7%; 17-23%)<br>176 (26.5%; 23-30%)<br>136 (20.5%; 17-24%)<br>128 (19.2%; 16-22%)<br>94 (14.1%; 12-17%)	103 (39.0%; 33-45%)<br>67 (25.4%; 20-31%)<br>39 (14.8%; 11-20%)<br>22 (8.3%; 5.3-12%)<br>33 (12.5%; 8.8-17%)	624 (28.5%; 27-30%)<br>653 (29.9%; 28-32%)<br>362 (16.6%; 15-18%)<br>279 (12.8%; 11-14%)<br>269 (12.3%; 11-14%
**Gender:** Female	236 (35.5%; 32-39%)	111 (42.0%; 36-48%)	902 (41.2%; 39-43%)
**Nutrition Status**<br>Well nourished<br>Mildly Malnourished<br>Moderately Malnourished<br>Severely Malnourished	252 (43.3%; 39-47%)<br>208 (35.7%; 32-40%)<br>71 (12.2%; 9.7-15%)<br>51 (8.8%; 6.6-11%)	102 (46.6%; 40-53%)<br>69 (31.5%; 25-38%)<br>27 (12.3%; 8.3-17%)<br>21 (9.6%; 6.0-14%)	833 (41.6%; 39-44%)<br>703 (35.1%; 33-37%)<br>287 (14.3%; 13-16%)<br>180 (9.0%; 7.8-10%)
**Vaccination History:** (Received BCG, Penta 1 and Penta 3)	617 (92.8%; 91-95%)	228 (86.4%; 82-90%)	2,055 (94.0%; 93-95%)
**Malaria Diagnosis**	13 (2.0%; 1.0-3.3%)	34 (12.9%; 9.1-18%)	874 (40.0%; 38-42%)
**SCD**	-	10 (3.8%; 1.8-6.9%)	351 (16.0%; 15-18%)
**Admission Day:** Weekday	505 (75.9%; 73-79%)	205 (77.7%; 72-83%)	1,716 (78.5%; 77-80%)
**Season:** Wet	368 (55.3%; 51-59%)	147 (55.7%; 49-62%)	1,129 (51.6%; 50-54%)
***Pf*PR$_{2-10}$** % Median (IQR)	34 (30, 46)	31 (29, 40)	33 (29, 46)
**Residence type:** Rural	503 (75.6%; 72-79%)	166 (62.9%; 57-69%)	1,548 (70.8%; 69-73%)

Nutrition status: n = 73 admissions were missing MUAC for those underlying conditions excluded. There are no counts for SCD for underlying conditions as SCD is an underlying condition that was included in the analysis. Nutrition status: n = 45 admissions were missing MUAC for those missing Hb.

SUPPLEMENTARY NOTES 2

Travel time to emergency care services:

Time taken to travel from the patient's village (EA) of residence was calculated using a least cost path modelling approach in AccessMod software (alpha version 5.8.0)(1). The modeling process utilized auxiliary datasets detailed as follows.

a) *Roads Network*: Road networks received from Kenya Roads Board (KRB) 2021 were cleaned and reclassified into five classes; international and national trunk roads, primary and secondary roads, minor and government roads, settlement roads and rural access farm and unclassified roads (Table S2, Figure S1a).

b) *Land Use/Cover*: Given that not all areas are covered by roads or footpaths, satellite-derived information from the European Space Agency (ESA) Sentinel-2 imagery 2021 was used to classify the nature of the underlying geographic space covered by patients on their way to hospital in the absence of roads. The $10\text{m} \times 10\text{m}$ land cover obtained from ESA had eight classes: tree cover, shrubland, grassland, cropland, built-up areas, bare and sparse vegetation, waterbodies and herbaceous wetlands (Table S2, Figure S1b).

c) *Travel Barriers*: Rivers, national parks and game reserves were considered as barriers to travel. Although lakes are present in the region, lakes were not included as barriers since water transport in the form of boats is present. Data on barriers to transport were obtained from world database on protected areas.

d) *Digital Elevation model (DEM)*: Both walking and cycling speeds are significantly impacted by varying slopes of the land. To ensure the variations in the speeds are accounted for in the estimation of travel time, the digital elevation model was obtained from ALOS PALSAR at $12.5\text{m} \times 12.5\text{m}$ spatial resolution.

e) *Travel Scenario*: The travel scenario adopted incorporated walking, motorcycle and vehicle transportation based on the different land cover types (Table S2). Therefore, based on the fastest route to BCRH, an individual could walk to BCRH if the fastest path does not include any motorised land cover category or use sequences of the different transportation modes based on the land cover categories that constitute the fastest path to BCRH.

Table S2: Land cover classifications, Associated speeds and Modes of Transport

Land Cover Category	Speed (Km/Hr)	Mode of Transport
International and national trunk roads	65.00	Motorized
Primary and Secondary roads	40.00	Motorized
Minor and government roads	25.00	Motorized
Settlement roads	20.00	Motorized

Rural access farm and unclassified roads	20.00	Motorized
Tree Cover	2.50	Walking
Shrubland	5.00	Walking
Grassland	3.50	Walking
Cropland	3.25	Walking
Built-Up	5.00	Walking
Bare and Sparse Vegetation	5.00	Walking
Waterbodies	8.50	Motorized
Herbaceous Wetland	2.00	Walking

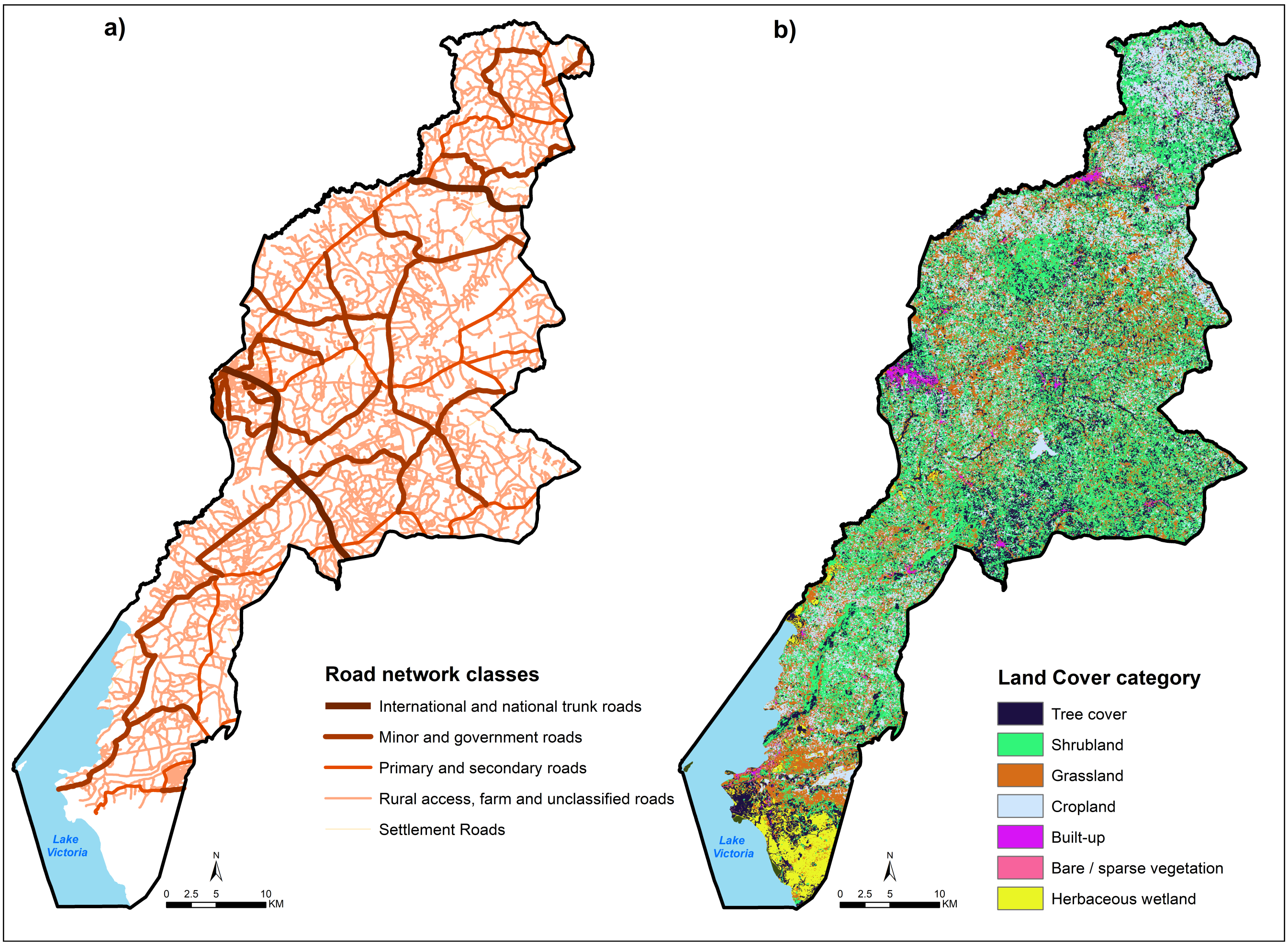

Figure S1: Spatial Distribution of **Panel a):** Road network and **Panel b):** Land cover categories used in the calculation of travel time.

The ”Accessibility module” in AccessMod software (alpha version 5.8.0) was implemented to model travel time to Busia County referral hospital (https://www.accessmod.org/). The algorithm in AccessMod models travel time using the terrain-based least cost path distance calculation and is widely applied in the computation of spatial access metrics (2). To model travel time,

a) a friction raster surface was created using the road, land cover and travel barriers datasets described above under the ”merge land cover” module in AccessMod.

b) Motorised and walking speeds were assigned to the different road classes and land cover types as shown in Table S2 based on previous literature (3; 4; 5). The study assumed that a care-seeker takes the pathway with the lowest travel time to BCRH. In this pathway, a care-seeker would walk if above a land cover assigned walking mode of transport, use a motorcycle if above a land cover assigned motorised mode of transport (Table S2). In addition, the care-seeker can switch from one mode of transport to another based on the land cover types that constitute the fastest path to BCRH.

c) DEM was used to account for changing walking speeds due to variations in slope according to Tobler’s formulation; an exponential function that describes how human walking speed varies with slope (1).

d) Travel time was then be computed, i.e., AccessMod calculated the travel time towards the hospital. The output from AccessMod was a raster surface of the travel time of each pixel to the hospital (Figure S5a).

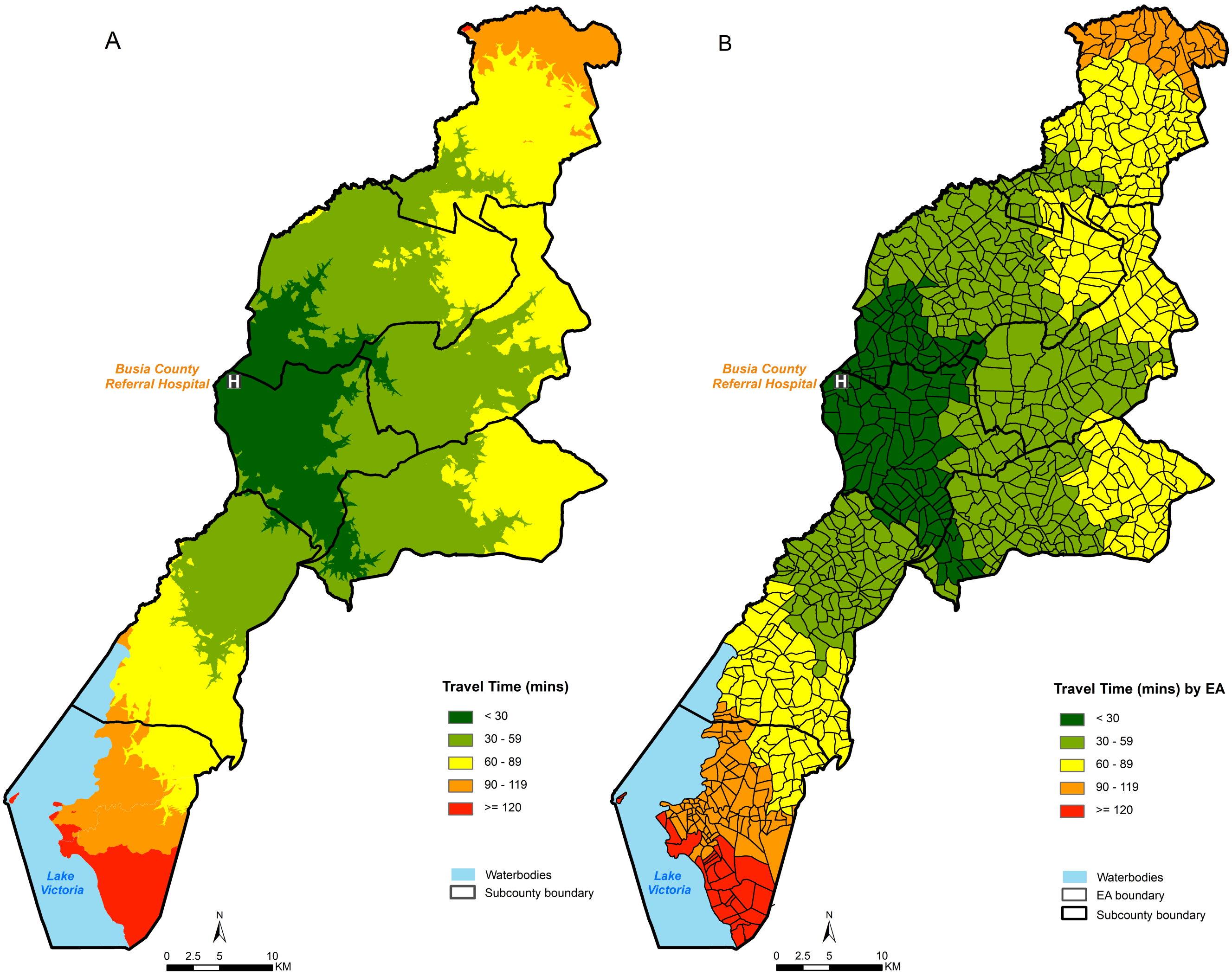

Figure S2: Spatial Distribution of Travel time in Busia County. **Panel a):** 12.5 m × 12.5 m travel time raster from AccessMod visualized by 30-minute time bands. **Panel b):** Average travel time by EA extracted from the travel time raster and visualized by 30-minute time bands.

The travel time for each EA was extracted using the extract function under the terra package in R software version 4.4.0 (2024-04-24 ucrt). The extract function requires three arguments; the raster data to extract from, the extent of the extraction and the function to extract the values. The travel time raster from AccessMod and the EA shapefile were the respective inputs - travel times were extracted as the average values of each EA (Figure S5b). Each admission was then assigned the travel time corresponding to their EA of residence.

SUPPLEMENTARY NOTES 3

Plasmodium falciparum Prevalence Rate (*Pf*PR$_{2-10}$)

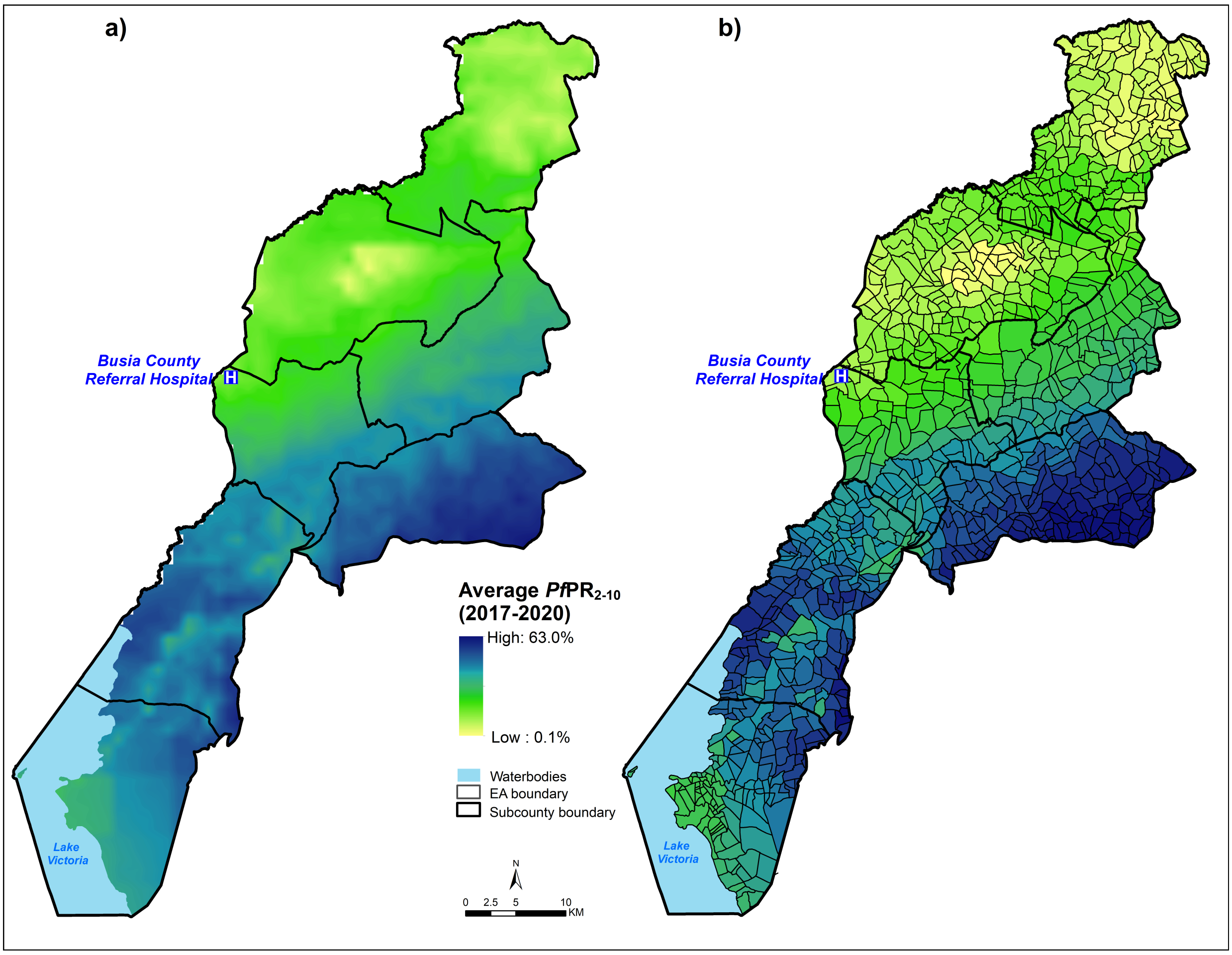

Figure S3: Spatial Distribution of average *Pf*PR$_{2-10}$ for the years 2017-2020. **Panel a)**: *Pf*PR$_{2-10}$ raster obtained from averaging individual annual *Pf*PR$_{2-10}$ rasters (2017-2020) obtained from Alegana et al (6). **Panel b):** Average *Pf*PR$_{2-10}$ per EA extracted from the *Pf*PR$_{2-10}$ raster (Panel a). In brief, Alegana et al (6) assembled malaria prevalence data for Kenya, mainland Tanzania and Uganda from annual malaria surveys for the period January 2010 – June 2020 and fitted a Bayesian spatiotemporal model adjusting for population and ecological/environmental covariates to interpolate the annual community's risk of malaria, *Plasmodium falciparum* Prevalence Rate, at a fine geographical scale for the years 2010-2020.

SUPPLEMENTARY NOTES 4

Selection of Effect Modifiers/covariates and Model Diagnostics

Table S3: Univariate analysis on the risk of severe anaemia over other anaemia classes

Characteristic	Crude Odds Ratio (95% CI)	p-value
**Age Categories (yrs)**		
<1	Ref	< 0.001
1	0.94 (0.72 - 1.23)	
2	1.99 (1.49 - 2.65)	
3	2.23 (1.64 – 3.04)	
4	3.16 (2.33 - 4.30)	
**Gender:**		
Male	Ref	0.4
Female	1.08 (0.90 - 1.30)	
**Nutrition Status**		
Well nourished	Ref	0.071
Mildly Malnourished	1.00 (0.78 - 1.28)	
Moderately Malnourished	0.79 (0.62 - 0.99)	
Severely Malnourished	1.04 (0.84 - 1.29)	
**Vaccination History:**		
No	Ref	0.094
Yes	1.42 (0.94 - 2.18)	
**Malaria Diagnosis**		
No	Ref	< 0.001
Yes	2.23 (1.85 - 2.69)	
**SCD**		
No	Ref	< 0.001
Yes	2.11 (1.67 - 2.68)	
**Admission Day:**		
Weekend	Ref	< 0.009
Weekday	1.36 (1.08 - 1.73)	
**Season:**		
Dry	Ref	< 0.2
Wet	0.9 (0.75 - 1.08)	
***Pf*PR$_{2-10}$**	1.04 (1.03 - 1.05)	< 0.001
**Residence type:**		
Urban	Ref	< 0.001
Rural	3.83 (2.98 - 4.97)	

Gender and season did not meet the $P < 0.2$ threshold (Table S3) and were excluded from the final model.

SUPPLEMENTARY NOTES 5

Evidence for Spatial Autocorrelation

Prior to fitting spatial models under the INLA-SPDE framework to assess association between travel time to EC services and severe anaemia, the presence of spatial autocorrelation was investigated using a variogram based on residuals; a standard tool for assessing presence of spatial autocorrelation (7). Supplementary Figure 3 shows evidence for the existence of spatial dependence in the dataset. From Supplementary Figure 3, there exists spatial dependence up to a range of ≈ 50 km, thus, the spatial model is justified.

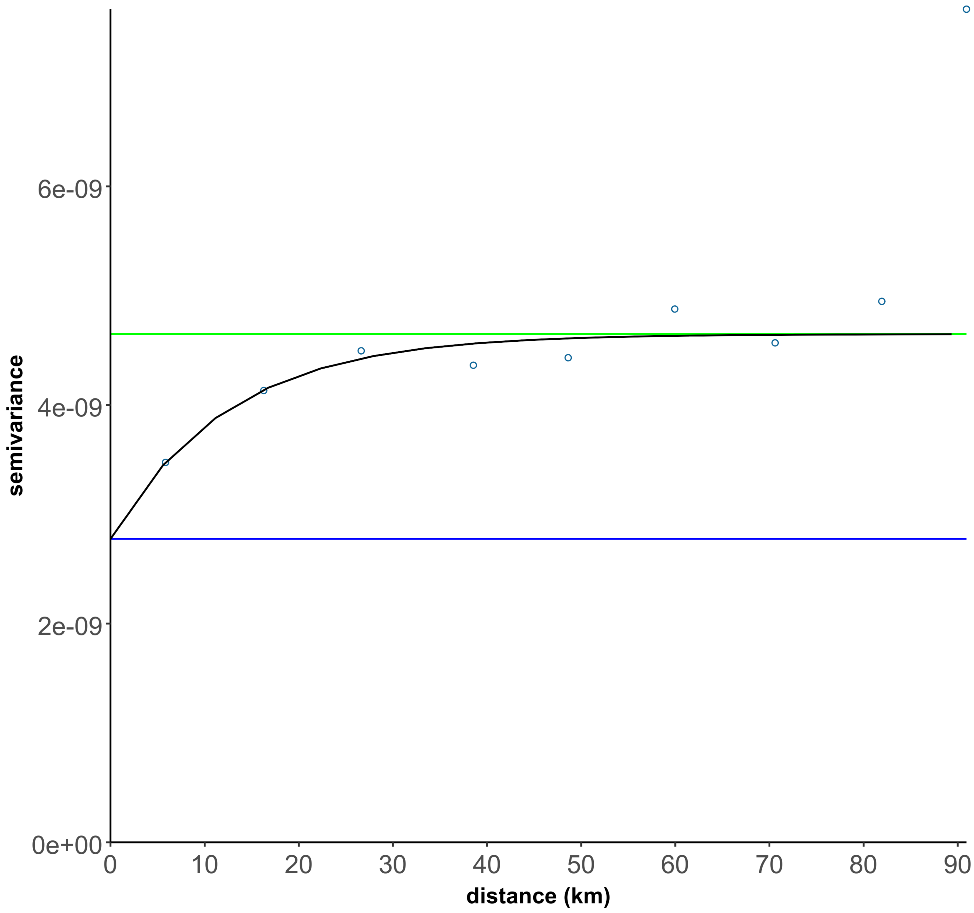

Figure S4: Variogram - Evidence for Spatial Dependence

SUPPLEMENTARY NOTES 6

Table S4: Association between covariates among children with severe anaemia compared to other (mild/moderate) anaemia

Characteristic	Adjusted Odds Ratio (95% CI)
**Age Categories (yrs)**	
<1	Ref
1	0.72 (0.52 - 0.99)
2	1.11 (0.78 - 1.57)
3	1.13 (0.78 - 1.64)
4	1.47 (1.01 - 2.13)
**Nutrition Status**	
Well nourished	Ref
Mildly Malnourished	1.08 (0.82 - 1.44)
Moderately Malnourished	0.9 (0.69 - 1.17)
Severely Malnourished	1.01 (0.79 - 1.28)
**Vaccination History:**	
No	Ref
Yes	0.44 (0.21 - 0.92)
**Malaria Diagnosis**	
No	Ref
Yes	2.97 (2.29 - 3.85)
**SCD**	
No	Ref
Yes	2.95 (2.14 - 4.07)
**Admission Day:**	
Weekend	Ref
Weekday	1.35 (1.03 - 1.76)
***Pf*PR$_{2-10}$**	2.53 (1.41 - 4.46)
**Residence type:**	
Urban	Ref
Rural	1.55 (0.81 - 2.85)

Adjusted OR: are from multivariate analysis fitted under the Bayesian MBG framework adjusting for the covariates and spatial autocorrelation.

SUPPLEMENTARY NOTES 7

Travel time to nearest lower-level facility

Using the approach described under supplementary notes 2, travel times to the nearest lower-level public health facility with provisions for paediatric admissions was calculated (Supplementary Figure 5).

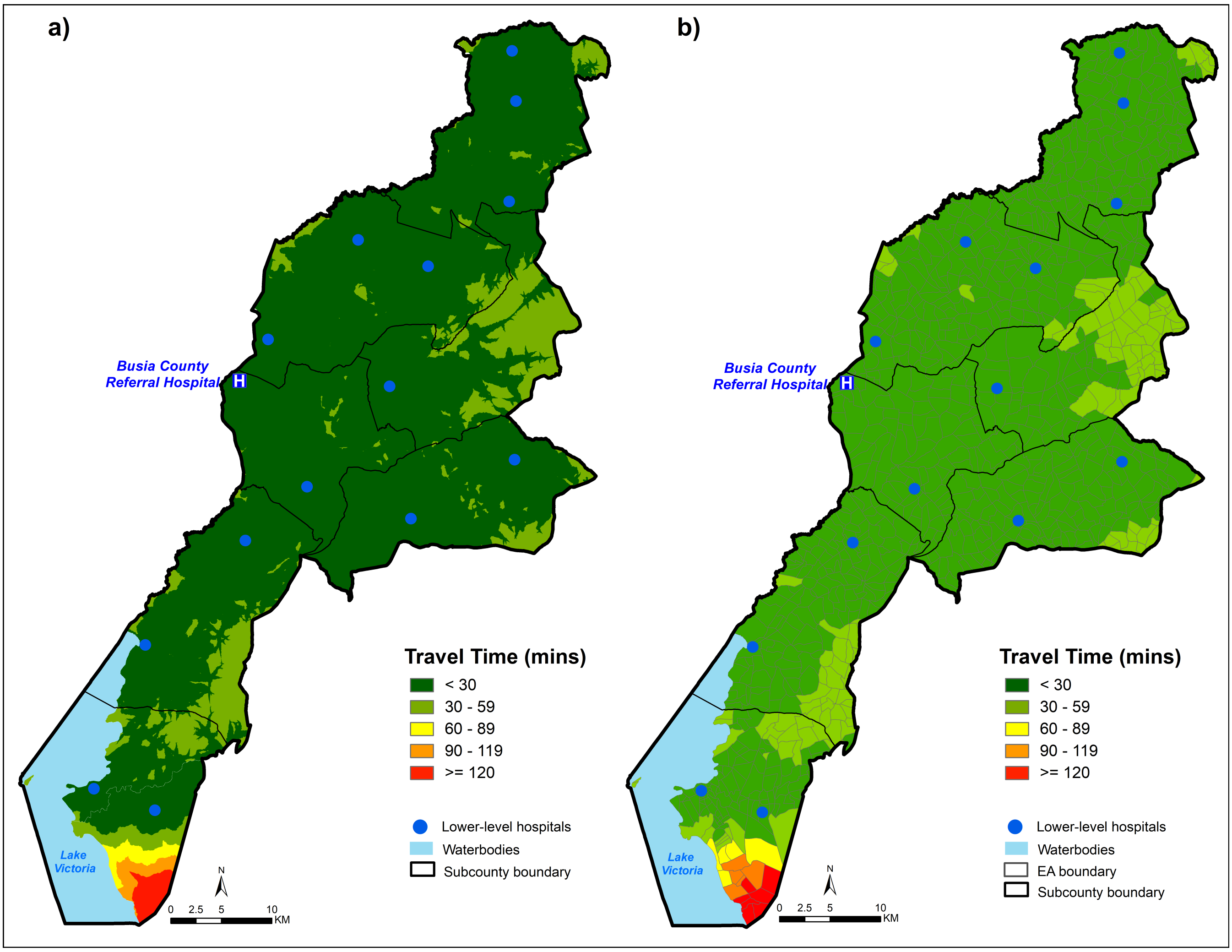

Figure S5: Spatial distribution of travel time to nearest lower-level public health facility with provisions for paediatric admissions in Busia County. Panel a): 12.5 m $\times$ 12.5 m travel time raster from AccessMod visualized by 30-minute time bands. Panel b): Average travel time by EA extracted from the travel time raster and visualized by 30-minute time bands.

If the lower-level facility provided EC services for severe anaemia, the distribution of EAs across travel time bands would be as follows: 860 EAs within less than 30 minutes, 110 EAs within 30-59 minutes, 5 EAs within 60-89 minutes, and 14 EAs within $\geq$ 90 minutes. This distribution supports our conclusion; it is indicative of marked improvement in travel times as currently, the distribution of EAs as per travel time to BCRH is 126 EAs within less than 30 minutes, 406 EAs within 30-59 minutes, 303 EAs within 60-89 minutes, and 154 EAs within $\geq$ 90 minutes.

REFERENCES

[1]Ray N, Ebener S. AccessMod 3.0: computing geographic coverage and accessibility to health care services using anisotropic movement of patients. Int J Health Geogr. 2008 12;7:63.

[2]Bouanchaud P, Macharia PM, Demise EG, Nakimuli D. Comparing modelled with self-reported travel time and the used versus the nearest facility: modelling geographic accessibility to family planning outlets in Kenya. BMJ Global Health. 2022 5;7:e008366.

[3]Dixit A, Lee MC, Goettsch B, Afrane Y, Githeko AK, Yan G. Discovering the cost of care: consumer, provider, and retailer surveys shed light on the determinants of malaria health-seeking behaviours. Malaria Journal. 2016 Mar;15(1).

[4]Joseph NK, Macharia PM, Ouma PO, Mumo J, Jalang'o R, Wagacha PW, et al. Spatial access inequities and childhood immunisation uptake in Kenya. BMC Public Health. 2020 9;20:1407.

[5]Macharia PM, Mumo E, Okiro EA. Modelling geographical accessibility to urban centres in Kenya in 2019. PLOS ONE. 2021 May;16(5):e0251624.

[6]Alegana VA, Macharia PM, Muchiri S, Mumo E, Oyugi E, Kamau A, et al. Plasmodium falciparum parasite prevalence in East Africa: Updating data for malaria stratification. PLOS Global Public Health. 2021 12;1:e0000014.

[7]Bohling G. Introduction to geostatistics and variogram analysis. Kansas geological survey. 2005;1(10):1-20.
